# The acceptability to women in Mombasa, Kenya, of the donation and transfusion of umbilical cord blood for severe anaemia in young children

**DOI:** 10.1111/j.1423-0410.2007.01012.x

**Published:** 2007-12-07

**Authors:** O Hassall, L Ngina, W Kongo, J Othigo, K Mandaliya, K Maitland, I Bates

**Affiliations:** 1 Centre for Geographic Medicine Research (Coast), Kenya Medical Research Institute/Wellcome Trust Research ProgrammeKilifi, Kenya; 2 Liverpool School of Tropical MedicineLiverpool, UK; 3 International Centre for Reproductive HealthMombasa, Kenya; 4 Regional Blood Transfusion CentreMombasa, Kenya; 5 Coast Provincial General HospitalMombasa, Kenya; 6 Imperial College, South Kensington CampusLondon, UK

**Keywords:** acceptability, anaemia, child, consent, HIV, sub-Saharan Africa, umbilical cord blood

## Abstract

**Background and Objectives:**

Severe anaemia, for which a blood transfusion can be life saving, is common in hospitalized children in sub-Saharan Africa but blood for transfusion is often in short supply. Umbilical cord blood is usually thrown away but could be a useful source of red cells for small volume transfusions in young children in this setting. The objective of this study was to evaluate the attitudes of women using the maternity services of the provincial hospital in Mombasa, Kenya, towards cord blood donation and transfusion, and essential aspects of this process including informed consent and the acceptability of screening for human immunodeficiency virus (HIV) infection.

**Materials and Methods:**

A structured questionnaire was developed based on data provided by focus group discussions with women attending the hospital's maternity unit and administered to women who had recently delivered at the hospital.

**Results:**

Of the 180 women who completed a questionnaire, the donation and transfusion of cord blood were acceptable to 81% and 78%, respectively. Ninety per cent of women who supported cord blood donation were willing to undergo further HIV testing at the time of delivery. Seventy-seven per cent of women wanted informed consent to be sought for cord blood donation and 66% of these felt they could make this decision alone.

**Conclusion:**

The donation of umbilical cord blood and its transfusion are acceptable to the majority of women delivering at Coast Provincial General Hospital, Mombasa. Findings from the study will benefit the planned cord blood donation programme at this facility.

## Introduction

### Severe anaemia in children in sub-Saharan Africa

Severe anaemia is a major public health problem in sub-Saharan Africa and has been estimated to kill as many as 974 000 children under 5 years old per year [1]. In malaria endemic areas, *Plasmodium falciparum* malaria is the most common cause of severe anaemia in children admitted to hospital and children aged less than 24 months are the most frequently affected. The prevalence of severe anaemia (defined as haemoglobin < 5 g/dl) in hospitalized children is reported to range from 8 to 29% with case fatality rates of 8–18% [2].

In children with severe, uncompensated anaemia, blood transfusion can substantially reduce mortality [3]. Over 50% of deaths occur within 6 h of admission and early intervention and the ready supply of safe blood have been identified as key components in the hospital treatment of severe anaemia in childhood [4–6].

In many countries of sub-Saharan Africa, the administration of blood is often delayed because the blood supply is insufficient. This reflects the situation in all developing countries where there is an over-reliance on family/replacement donors and an estimated shortfall of 40 million units of blood a year [7,8]. In these circumstances, it has been proposed that umbilical cord blood (cord blood) could provide a useful supplementary source of blood for transfusion in young children [9,10].

### Attitudes towards blood in sub-Saharan Africa

In traditional societies in sub-Saharan Africa, blood may be associated with taboos [11]. Negative attitudes towards blood donation for transfusion are also recognized and these include anxieties concerning the risk of human immunodeficiency virus (HIV) infection [12–15]. Concerns have been expressed about blood sampling for research purposes in a coastal community in Kenya [16]. Cord blood is not routinely collected anywhere in sub-Saharan Africa and attitudes towards its donation and transfusion are unknown. Negative attitudes towards the process of cord blood collection and the concept of cord blood donation and transfusion would be a significant barrier to pursuing the use of cord blood as a supplementary source of blood for transfusion. Locally appropriate tools and local data are required to judge the acceptability of cord blood donation and transfusion among those populations whom it might benefit.

### Consent for cord blood donation

Important differences between routine blood donation and cord blood donation have consequences for the consent process for cord blood donation. These are described by Vawter *et al*. [17] and include: cord blood can be considered a waste product and consent for its collection may not be necessary; the donors are infants and cannot consent on their own behalf; there is only a single, short opportunity to donate; prior to the donation the mother is in labour and may be unable to sufficiently absorb and reflect on information to make an informed decision to donate; and, the risks of *ex utero* cord blood collection to the mother relate to the results of screening tests rather than the procedure itself.

One strategy that is used to overcome the complexities of informed consent for cord blood donation for stem cell banking in developed countries is a phased consent process. This consists of three stages: first, general information regarding cord blood collection and banking is provided during routine antenatal care; second, consent to collect cord blood is sought during labour; and third, further consent to screen, store and use the blood is sought after delivery and collection [17].

In addition to the peculiarities of cord blood collection common to any setting, there are additional factors that may be hypothesized to influence a consent process for cord blood collection in Kenya and other countries in sub-Saharan Africa. These include: the diversity of antenatal care providers, a high prevalence of HIV compared to developed countries, high rates of home delivery, late presentation for delivery care, rapid discharge after delivery to avoid user fees, exclusion of partners and family members from labour ward, poor recognition of patient autonomy and right to informed consent for routine clinical procedures, and the subordinate role of women in decision-making.

Given the likely high number of antenatal care providers and the low rates of hospital delivery, we did not consider universal antenatal information-giving (the first stage in the developed country model) to be feasible in this setting. But, we felt that a two-stage consent process using the second and third stages of the model described above could be of potential value. The key, however, to an appropriate informed consent process is a better understanding of local attitudes towards consent for cord blood donation for transfusion among potential cord blood donors.

### Serological screening and antenatal care

National Blood Transfusion Service policy in Kenya is to screen blood donations for HIV, hepatitis B, hepatitis C and syphilis. The higher the prevalence and incidence of HIV and other transfusion-transmitted infections in a blood donor population, the greater the wastage related to the discard of units that screen positive and the residual risk of a false-negative screening result. In a higher prevalence setting such as Kenya, a potential benefit of cord blood donation for transfusion is the opportunity of collecting cord blood from women who have already been recently screened for HIV and syphilis as part of their routine antenatal care. This advantage would be mitigated, however, if rates of antenatal screening are low or if a further test around the time of delivery is unacceptable to potential cord blood donors.

### Aims of the study

We plan to establish a cord blood donation programme at a site in sub-Saharan Africa in order to assess the bacterial and virological safety of cord blood in this setting. The aim of this study was to evaluate the acceptability and feasibility of cord blood donation and transfusion to potential cord blood donors at the proposed study site. The primary objective was to establish the proportion of women for whom cord blood donation for transfusion is acceptable. Secondary objectives were: to establish the proportion of women for whom cord blood transfusion for a child is acceptable; to quantify attitudes towards consent for cord blood donation in general and a two-stage consent process in particular; and to determine the rates of antenatal HIV testing and the acceptability of further HIV testing as part of a transfusion-transmitted infection screen of cord blood.

## Methods

### Study site

The study was undertaken between April and September 2005 at Coast Provincial General Hospital (CPGH), Mombasa, which is a large government referral facility in an urban setting. Annually, the hospital manages approximately 10 000 deliveries, 40 000 admissions and 1000 paediatric transfusions. The HIV prevalence in the antenatal population in Mombasa is about 10% (International Centre for Reproductive Health, unpublished data).

### Research tool

The research tool used was a structured questionnaire with the range of views regarding cord blood collection, cord blood transfusion and consent, established inductively *a priori* by focus group discussion [18].

Eight focus group discussions were convened and participants were recruited from women attending CPGH for antenatal services or who had recently delivered at CPGH and were awaiting discharge. Each focus group had 8–10 participants and sampling was purposive with particular reference to religion, tribal affiliation (community) and parity. A discussion guide was developed and minor modifications were made after piloting. The discussions were facilitated in Kiswahili by a female Kenyan (LM), tape-recorded and then translated into English, transcribed and verified. Content analysis of the transcripts was undertaken using a constant comparison approach with the assistance of software designed for the purpose (Nvivo v2·0, QSR pty).

The themes that emerged from the focus group discussions with regard to attitudes towards cord blood donation, cord blood transfusion and consent were developed into the structured questionnaire, which also included questions relating to biographical information and antenatal experience, including HIV testing. There was no direct question about HIV status as we felt that this could undermine trust between questioner and respondent and yield little in terms of the study objectives.

The questionnaire was translated into Kiswahili and piloted with 15 eligible respondents (10% of sample size), sampled opportunistically from the maternity unit at CPGH. As a result, minor changes were made to syntax and language and these adjustments were tested by piloting with a further 15 respondents.

During the focus group discussions, it was clear that for some of the participants there was initial misunderstanding about the origin and nature of cord blood. In particular, there was confusion between cord blood and the blood-stained liquor seen at delivery and in some cases this was thought to be menstrual loss accumulated over the gestation period. The fetal, as opposed to maternal, origin of cord blood was also sometimes poorly appreciated. As a consequence, we developed a set of line drawings to illustrate our definition of cord blood.

The structured questionnaire was administered individually to women who had recently delivered a living infant at CPGH, before their discharge from the hospital. At the beginning of the interview the following concepts were introduced: the origin and nature of cord blood (using pictures); cord blood donation and allogeneic cord blood transfusion; and blood donor screening for HIV, hepatitis and syphilis. Interviews were conducted in private, questions were read out in Kiswahili and responses were recorded by a trained fieldworker.

### Sampling and statistical methods

The sample size for the questionnaire was based on the primary objective, which was to establish the proportion of women delivering at CPGH for whom cord blood donation was acceptable. From responses during the focus group discussions, we estimated that this proportion was likely to be in the region of 60% and calculated that a minimum sample size of 150 would give acceptable precision with 95% confidence intervals of ±8%. Sampling was opportunistic, with all eligible women approached during normal working hours in a 4-week period in September 2005.

Data from completed questionnaires were entered onto a database (Epidata v3·1) and were analysed using EpiInfo (v6·02). Binary data were expressed as proportions (with 95% confidence intervals where appropriate), and observed differences compared for statistical significance with the χ^2^-test of association. Non-normally distributed data were summarized by the median and compared for statistically significant differences by the Wilcoxon rank-sum test.

### Ethics

Participation in the focus group discussions and questionnaire survey was voluntary and individual respondents were fully informed of the nature of the study and gave their verbal consent. The study was approved the Kenya National Ethics Committee and the CPGH Ethics Committee.

## Results

Of the 515 women who delivered at CPGH during the study period, 187 (36%) were approached to respond to the structured questionnaire. Four women declined to participate (98% response rate) and three questionnaires were completed incorrectly; therefore, 180 questionnaires were included in the analysis. Demographic and obstetric characteristics of the respondents are shown in Table 1.

**Table 1:** Demographic and obstetric characteristics of respondents stratified by attitude towards cord blood donation and transfusion

		Cord blood donation		Cord blood transfusion	
			
	All respondents	Acceptable	Unacceptable	Acceptable	Unacceptable
N (%)	180	146 (81%)	34 (19%)	141 (78%)	39 (22%)
95% CI		(75%–87%)	(14%–25%)	(72%–84%)	(16%–28%)
Demographic characteristics					
Age (mode)	20–24	20–24	25–29	20–24	25–29
≤ 24 years	98 (54%)	83 (57%)	15 (44%)	81 (57%)	17 (44%)
No formal education	17 (10%)	12 (8%)	5 (15%)	16 (11%)	1 (3%)
Some/completed primary education	91 (51%)	73 (50%)	18 (53%)	70 (50%)	21 (54%)
Secondary education	72 (40%)	61 (42%)	11 (32%)	55 (39%)	17 (44%)
Married	159 (88%)	126 (86%)	33 (97%)	123 (87%)	36 (92%)
Muslim	51 (28%)	42 (29%)	9 (26%)	44 (31%)	7 (18%)
Pentecostal/Independent Christian	87 (48%)	69 (47%)	18 (53%)	66 (47%)	21 (54%)
Employed	73 (41%)	60 (41%)	13 (38%)	56 (40%)	17 (44%)
Coastal tribe (Mijikenda)	54 (30%)	44 (30%)	10 (29%)	45 (32%)	9 (23%)
Obstetric history and antenatal experience					
Parity (median)	2	2	2	2	1
(range)	1–8	1–8	1–6	1–7	1–8
Previous hospital deliveries (median)	1	1	1	1	1
(range)	0–6	0–6	1–4	1–6	0–4
Caesarean section this delivery	24 (13%)	17 (12%)	7 (21%)	18 (13%)	6 (15%)
Antenatal care at CPGH	33 (18%)	28 (19%)	5 (15%)	28 (20%)	5 (13%)
Antenatal HIV test	127 (71%)	105 (72%)	22 (65%)	99 (70%)	28 (72%)

### Cord blood donation

Cord blood donation for transfusion was acceptable to 81% of respondents (146/180, 95% confidence interval: 75% to 87%). There were no statistically significant differences, with regard to demographic and obstetric characteristics, between those women who were willing for cord blood to be collected for transfusion and those who were not (Table 1).

The two major reasons (multiple responses permitted) stated for the acceptability of cord blood donation were: ‘To save a life’ (82%: 119/146) and ‘Because it is going to be thrown away anyway’ (32%: 47/146). Of the 14 respondents who specified a reason for cord blood donation other than those listed, ten stated that a motivation for donating cord blood was to know more about their own health status through screening tests, with particular reference to HIV. Eighty-nine per cent (130/146) of those willing to donate cord blood for transfusion would agree to have an HIV test at, or around, the time of delivery.

For the 19% of respondents (34/180) who were unwilling to donate cord blood for transfusion, the main reason given was that they considered the blood to be unsuitable for transfusion, because it is ‘unclean/dirty’, ‘worn out/weak’ or ‘not safe (contaminated)’ (29%: 10/34). Beliefs that cord blood donation for transfusion would be against faith (2/34: a Sunni Muslim and a Pentecostal Christian) or custom (1/34: a Luo from western Kenya), and fears of ‘witchcraft/Satanism’ (4/34), were not widely held.

Of those women unwilling to donate cord blood for transfusion, three gave the specific reason that ‘I don't want to undergo another HIV test’ and only 65% (22/34) would agree to have an HIV test at, or around, the time of delivery. This proportion was significantly less than in those women willing to donate cord blood for transfusion (22/34 vs. 130/146: χ^2^, *P* < 0·001).

### Cord blood transfusion

The receipt of a cord blood transfusion by their own child was acceptable to 78% of the respondents (141/180, 95% confidence interval: 72% to 84%) and there were no statistically significant differences in demographic and obstetric characteristics compared to those who found cord blood transfusion unacceptable (Table 1). Of those that found cord blood transfusion acceptable, 91% (128/141) were also willing to donate cord blood whereas in those that found cord blood transfusion unacceptable 46% (18/39: χ^2^, *P* < 0·001) were willing to donate cord blood.

The two main views (multiple responses permitted) associated with an indifference to the source of blood for transfusion were: ‘It does not matter as long as it has been tested [for transfusion transmissible infections and/or blood group]’ (73%; 103/141) and ‘[It does not matter] if it will save my child's life’ (57%; 81/141).

A cord blood transfusion for their own child was unacceptable to 39 (22%) of the respondents, of whom 14 (36%) felt the blood to be ‘contaminated’ or ‘unclean/dirty’. Two respondents felt that the transfusion of cord blood to be against their faith (a Pentecostal Christian and a Jehovah's Witness), and none felt it was against their community's customs. The proportion of women prepared to have an HIV test at, or around, the time of delivery was significantly greater in those who found cord blood transfusion acceptable compared to those that did not (124/141 vs. 28/39: χ^2^, *P* = 0·014).

### Consent

Of the 180 respondents, 77% (139/180, 95% confidence interval: 70% to 83%) felt that informed consent should be sought for the collection of cord blood at a hospital delivery. The main reason given being ‘I have a right to know what is happening to my body’ (88%; 123/139). The proportion of women who thought a consent process necessary was similar irrespective of differing views concerning the acceptability of cord blood donation and transfusion.

Twenty-three per cent of women (41/180) felt that informed consent was not necessary for cord blood collection. For 61% of these (25/41) this was because the cord blood would be ‘discarded anyway’, and for 37% (15/41) because they were ‘only interested in the baby, so I don't care what happens to the cord’. Three respondents felt that informed consent was unnecessary because the blood would be ‘taken no matter what I say’.

Of the 139 women who felt that informed consent should be sought before cord blood collection, the majority (66%: 92/139) considered that the mother alone should give/refuse consent. A minority felt that it should be the father (14%: 20/139), or the mother and father together (11%: 15/139). Ten respondents felt that the hospital should be approached for consent on their behalf. Excluding these 10 women, 57% (73/129) of respondents who felt that consent was necessary considered verbal consent sufficient. The remainder (43%; 56/129) thought there should be a signed agreement.

For the 129 women who felt that they and/or a relative should give consent, the location of preference for a single informed consent event was: the antenatal clinic 25% (32/129), the labour ward before delivery 19% (24/129), the labour ward after delivery 25% (32/129), and the postnatal ward 32% (41/129). When all women who considered consent necessary for cord blood donation were asked specifically about a two-stage phased consent process, 89% (124/139) thought it acceptable.

### Antenatal experience and human immunodeficiency virus screening

Of the 180 respondents, 96% (173/180) had attended an antenatal clinic at least once during their pregnancy. Fifty-five per cent of these (95/173) received their antenatal care from five antenatal clinics, of which the largest proportion had attended the antenatal clinic at CPGH (35%: 33/95). The remaining 78 women had attended 44 different municipal and private clinics in Mombasa District and beyond.

Of the 173 women who had attended an antenatal clinic, 85% (147/173) had been offered an HIV test as part of their antenatal care and, of these, 127 (73%; 127/173) had undergone testing. Thus, 71% (127/180) of all respondents had had an antenatal HIV test and a further 16 women stated that they already knew their status from a previous test. We did not seek to ascertain the HIV status of any of the women in this study but we estimate, using the antenatal prevalence rate of 10%, that 14 women in this sample may have known that they were HIV positive.

Twenty-nine per cent (53/180) of the respondents had not had an HIV test during their preceding pregnancy, because they had not attended an antenatal clinic (seven); they were not offered or did not know if they had been offered an HIV test (26); or they were offered a test but chose not to be tested (20). Three of the latter stated that they already knew their HIV status. Of the 53 women who had not had an HIV test as part of their antenatal care, 81% (43/53) would take up an opportunity for counselling and testing on the labour ward if it were offered.

## Discussion

Cord blood donation for transfusion is acceptable to the majority of women who deliver at CPGH, and most would donate through altruistic intent. This is consistent with qualitative data concerning cord blood donation in Ghana [19], and attitudes towards blood donation in general [20].

Our data show no association between positive or negative views on cord blood donation and easily derived demographic or obstetric information, such as faith, tribal affiliation or parity. Such factors cannot, therefore, be used to screen for individuals more likely to find cord blood donation acceptable. Views about HIV and HIV testing, however, do seem to be important in determining the acceptability of cord blood donation and transfusion but they are contradictory. In our sample, HIV testing as part of a transfusion-transmitted infection screen for a cord blood collection would motivate some mothers to donate cord blood but deter others.

As the opportunity for testing for HIV and other transfusion-transmitted infections is a positive factor for some women who find cord blood donation acceptable, a cord blood collection programme should be able to provide test results and post-test counselling.

One deterrent to a further HIV test might be known HIV seropositivity. Mothers who know they are HIV positive might not want or see the need for a further HIV test and, knowing they are unsuitable donors, consider cord blood donation unacceptable. This could explain the correlation we observed between unwillingness to have a further HIV test and the unacceptability of cord blood donation. This hypothesis cannot be tested with our data, as we did not seek to know the HIV status of any of the women in the study. We consider, however, that if this behaviour is likely, consent to collect cord blood should be sought in a sensitive and confidential manner such that self-exclusion is encouraged and stigmatization minimized.

The fears of those women who feel cord blood donation and transfusion to be unacceptable because it is too dirty, worn out or contaminated may relate to factors other than HIV. These fears may be allayed by further development of information materials illustrated with diagrams describing the role and origin of cord blood and differentiating it from, for example, liquor or menstrual loss. This is perhaps of more relevance in this setting than in developed nations where education levels are higher and more prominence is given to health education during antenatal care.

In our sample of potential cord blood donors, the majority felt that it would threaten their autonomy if cord blood was collected without consent, and this is consistent with current guidelines for cord blood collection for stem cell banking in developed countries [17]. The fact that some women felt that their views were irrelevant, and others considered that the ‘hospital’ could make the decision for them is perhaps evidence of the paternalism extant in health systems in developing countries, or the subordinate role of women in decision-making in this society. It does, however, contrast with the view held by the majority of women who felt consent was necessary and that the mother could make the decision alone without deferring to the hospital, her husband or another family member. This is important for future cord blood collection at CPGH as relatives are generally excluded from the labour ward and routinely seeking additional consent from them would be challenging.

The high acceptability of our proposed two-stage consent process for cord blood donation, screening and transfusion is encouraging. The high number, diversity and dispersion of clinics, which provided antenatal services to the women in the survey confirms our view that an antenatal sensitization stage of a phased consent process as part of ‘routine’ antenatal care would present considerable logistical challenges in terms of distribution of materials, staff training and supervision. While widespread sensitization of pregnant women to cord blood donation should remain an aspiration, a more pragmatic approach might be to initially concentrate effort and resources on the five facilities with which just over half the women had had contact. Other forms of communication, such as the print and broadcast media (particularly local radio), should also be considered.

The feasibility of cord blood collection for transfusion at CPGH is enhanced by the high proportion of women delivering there who have attended an antenatal clinic and had a recent HIV test. This is also encouraging for organizations providing antenatal and Prevention of Mother to Child Transmission services in Mombasa District. Conversely, 28% of women stated that they had not had an HIV test as part of their antenatal care and efforts to improve this situation need to continue. Additionally, however, a cord blood donation programme could integrate with and strengthen on-site testing for mothers presenting to the labour ward without having had an HIV test.

### Study limitations

The sampling strategy used to select respondents was not random; rather, all eligible women were approached opportunistically during normal working hours but not outside these times and at weekends. Therefore, the women that were interviewed may not be representative of women delivering at CPGH. Unfortunately, the quality of hospital data is not sufficient to assess this by analysing the demographic and obstetric characteristics of non-participants.

In addition to cord blood donation, we also asked this sample of mothers their views on cord blood transfusion. We cannot say with certainty whether the views expressed here correlate with those of mothers whose children actually require a blood transfusion. It is unlikely that the users of paediatric and maternity services at the same hospital have different demographic characteristics but other factors could be important for the mothers of sick children.

Although the findings are encouraging, the extrapolation of these results to other parts of Kenya and sub-Saharan Africa with different cultural environments should be treated with some caution. The methodological tools, however, may well be used in other contexts.

## Conclusions and recommendations

We conclude from this study that cord blood donation and transfusion are acceptable at CPGH and consider this a sufficiently positive indication to pilot and evaluate a cord blood donation programme at this site. Many of the findings from this study, such as expectation of individual informed consent by mothers; the acceptability of a two-stage phased consent process; the need for further development of appropriate information, education and communication materials; and the provision of the results of transfusion transmitted infection screening and post-test counselling, will be incorporated into this programme.

If cord blood donation can be established at CPGH, we intend to undertake further studies to evaluate the bacterial and virological safety of cord blood in this setting.
